# Earlier shift in race pacing can predict future performance during a single-effort ultramarathon under sleep deprivation

**DOI:** 10.5935/1984-0063.20190132

**Published:** 2020

**Authors:** Allison J Brager, Sukru Demiral, John Choynowski, Jess Kim, Bill Campbell, Vincent F Capaldi, Guido Simonelli, Steve Hammer

**Affiliations:** 1 Walter Reed Army Institute of Research, Behavioral Biology - Silver Spring - MD - United States; 2 Academy of Wilderness Medicine, Fellow - Knoxville - TN - United States; 3 Indian River State College, Biology - Fort Pierce - FL - United States; 4 Center for Advanced Research in Sleep Medicine, Hopital du Sacre- Coeur de Montreal, CIUSSS du Nord-de-l’Ile-de-Montreal, Montreal, Canada

**Keywords:** Military Medicine, Sleep Deprivation, Circadian Rhythms, Physical Performance

## Abstract

**Objective:**

We constructed research camps at single-effort ultramarathons (50 and 100 miles) in order to study human endurance capabilities under extreme sleep loss and stress. It takes > 24h, on average, to run 100 miles on minimal sleep, allowing us to construct 24h human performance profiles (HPP).

**Methods:**

We collected performance data plotted across time (race splits) and distance (dropout rates; n=257), self-reported sleep and training patterns (n=83), and endpoint data on cardiovascular fitness/adaptation to total sleep deprivation and extreme exercise/stress (n=127).

**Results:**

In general, we found that self-reported napping was higher for 100-miler versus 50-miler runners and that ultra-endurance racing may possibly pre-select for early morning risers. We also compared HPPs between the first 50 miles completed by all runners in order to examine amplitude and acrophase differences in performance using a cosinor model. We showed that even though all runners slowed down over time, runners who completed a 100-miler ultramarathon had an earlier acrophase shift in race pace compared to non-finishers.

**Discussion:**

We were able to identify time-dependent predictions on overall performance under minimal sleep, warranting the ultramarathon athlete as a unique demographic for future study of sleep and chronobiological relationships in the real world.

## INTRODUCTION

History tells us that one of the first marathoners died at the finish. A Greek soldier ran ~ 26.2 miles from Marathon to Athens, Greece to announce defeat over Persia. Since Ancient Greece, running a marathon is commonplace. Every year, > 50,000 individuals run the New York City marathon. Most marathons occur in highly controlled environments with adequate first aid stations and first responders on duty. Weather conditions and terrain can induce physiological stress, but these variables can be controlled to an extent. The present study, instead, focuses on extreme cases of exercise-induced physiological stress in the ultramarathoner; an athlete who completes races > 26.2 miles (usually 50 - 100 miles) and runs for > 24h, on average, in order to finish. Under these circumstances, ultramarathoner must run across the night, consequently resulting in total sleep deprivation and/or significant sleep loss.

Research on ultramarathoners is limited, but burgeoning. Our small research community has identified several trait-like phenotypes of pain tolerance^1^, emotional regulation^11^, and habitual resting/sleep strategies in preparation for and across the course of ultramarathons^14^. Sleep is an anabolic process required as a result of wakefulness^(4,12,21 )^and therefore, it is not surprising that time for sleep was highly valued in a recent study of ultramarathoners competing in a multi-day race^14^. In this sample population of > 200 ultramarathoners, 73.9% reported good habitual sleep practices in preparation for an ultramarathon. 25% of ultramarathoners reported napping on work days and > 50% of ultramarathoners reported napping on non-work days. Few runners napped during ultramarathons < 36h in duration, but habitual napping was very common for races > 36h.

Although measurements of habitual resting/sleep strategies in ultramarathoners and athletes, in general, are largely self-report^8,13,14^, self-reported sleep can provide initial, macroscopic assessments of the physiological “set point” (i.e., hours) of sleep need for anabolic recovery. At present, the physiological “set point” of (self-report) sleep need for team and individual sport athletes is estimated at 8h^8,13^. The timing of sleep/activity cycles also underlies athletic capabilities. The time at which athletes compete and train can influence win-loss records^3^, injuries^3^, and individual performance^7^. In general, athletes perform better and are less likely to get injured in the late biological morning^3^ and late evening^3,7,16^, driven, in part, by rhythms of cortisol and norepinephrine tone^16^. It is also known that early morning chronotypes (i.e., larks) have higher pain tolerance^10^ and thermoregulatory control^17^ compared to late evening chronotypes (i.e., owls) under sleep loss, allowing “larks,” in theory, better equipped to handle the physiological demands of an ultramarathon.

Here, we expanded upon this burgeoning area of research by analyzing performance and physiological data collected across 24h from the ultramarathoner. The present study was a field study that minimally intervened with the operations of ultramarathons, ensuring that our data were ecologically valid. We also capitalized on the logistics of a single-effort 100-mile ultramarathon in order to have an internally controlled field study of total sleep deprivation under extreme conditions. Our primary aim was to dissect time-dependent patterns of human endurance capabilities under extreme homeostatic load (total sleep deprivation/sleep loss/thermoregulation) by creating 24h human performance profiles (HPP). Our 24h HPPs of ultramarathoners first reported here allowed us to predict the ability to finish a 100-mile race with finishers having an earlier acrophase shift in race pace, warranting the ultramarathon athlete as a unique demographic for future study of sleep and chronobiological relationships in the real world.

## METHODS

### Study participants

Up to 257 runners participated in our study. Runners registered for the Fort Clinch 50- and 100-miler Ultra (March 2016) and Florida Keys 50- and 100-miler Ultra (spring 2016). Average age was 43.2 + 12.5 years. 96% of runners traveled < 1 time zone to participate (Florida has a large ultramarathon community) and 0.5% of runners traveled > 6 time zones to compete.

### Self-reported sleep and training habits

Runners were given access to the survey through a Weblet site post-race (n=83 participated). De-identified responses were exported into MS Excel. The survey inquired about sleep/training schedule (e.g., self-reported sleep duration, rise time, bedtime and training time), frequency and reason for nighttime awakenings, frequency of using sleep aids, and frequency and duration of daytime naps. Data analyses were performed with SPSS ver. 21. Data are summarized in Table 1.

**Table 1:** Sleep and training habits of ultramarathoners.

	N	Mean ± SD	# of Athletes
Self-reported sleep	83	-	-
Total sleep time, training day	-	6.8 ± 0.2 h	-
Total sleep time, race day	-	6.1 ± 0.2 h	-
Daytime napping	-	0.7 ± 0.1 h	18*
Restless sleep	-	-	63*
Sleep medications	-	-	10
Sleep Efficiency (%)	-	92.04 ± 17.59	-
Self-reported training schedules	83	-	-
Early Morning (< 07:00h)	-	-	36
Mid Morning (> 09:00h)	-	-	25
Evening	-	-	22

* p < 0.05, 50 vs. 100-milers

### Cardiac measurements

As a physiological measure of overall fitness/adaptation to race demands, including > 24h extended wakefulness/sleep loss, standard 12-lead EKG and orthostatic blood pressure measurements were assessed 5 min pre-race (prior to extended wakefulness) and post-race (> 24h extended wakefulness; n=127) in 100-miler runners. From these direct measures, pulse pressure and mean arterial pressure were calculated, as well as changes in all measures pre- to post-race. Data analyses were performed with SPSS ver. 21.

### Race performance

**Figure 1:**
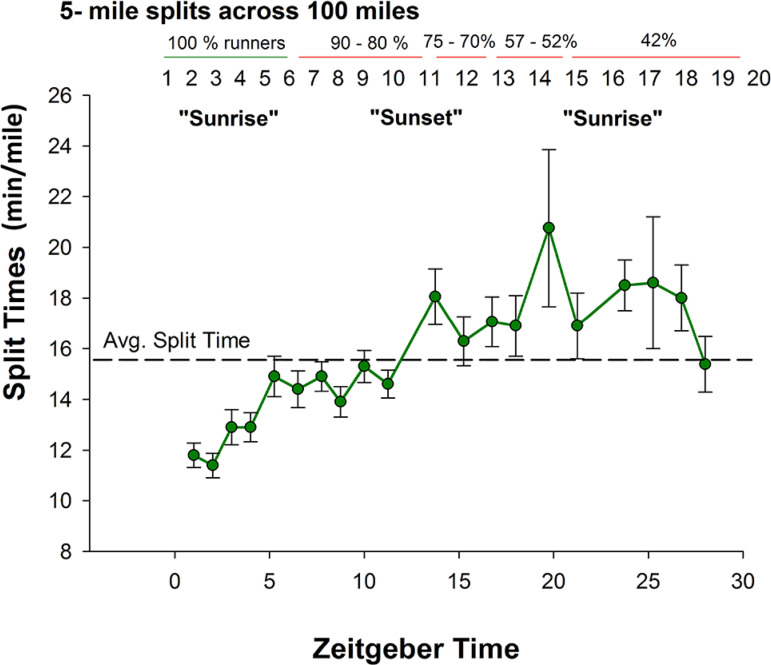
In order to dissect time-dependent patterns of human endurance capabilities under total sleep deprivation/sleep loss, running pace [green] was quantified every 5 miles of a 100-mile ultramarathon held in the spring (Amelia Island, FL; n=21, to start). Sample size across the race was dependent on dropout rate [upper X-axis, percentages; 100% [green]; < 100% [red]). Pace was under homeostatic influence for the first 50 miles and circadian influence from 50 - 100 miles. Race splits were plotted in Zeitgeber time (ZT; ZT 0/ZT 24, sunrise [~0730]; ZT 12, sunset [~1930]). The race started near sunrise (ZT 0/24). Average race pace was 15.1 + 2.3 min/mile. Average race finish time was 25.2 + 3.8h with continuous running efforts across the night (ZT 12 - ZT 24).

Distance split times and dropout rates were provided by race directors (n=257, in total) in order to perform general descriptive statistical analyses of race pace and overall finish time (mean *+* SE; SPSS ver. 21; *p*<0.05) of the Fort Clinch 50- and 100-miler Ultra (March 2016) and Florida Keys 50- and 100-miler Ultra (May 2016). For the Fort Clinch race (Amelia Island, FL), we were able to capture changes in race pace every 5 miles, allowing us to observe changes under homeostatic load (distance) and chronobiological influence (time) with high temporal resolution as shown in Figure 1. For the Florida Keys race, distance split times were more variable (i.e., every 5 miles, 10 miles, or 14 miles), allowing us to observe changes under homeostatic load (distance) and chronobiological influence (time) with only modest temporal resolution.

### Model fits

**Figure 2:**
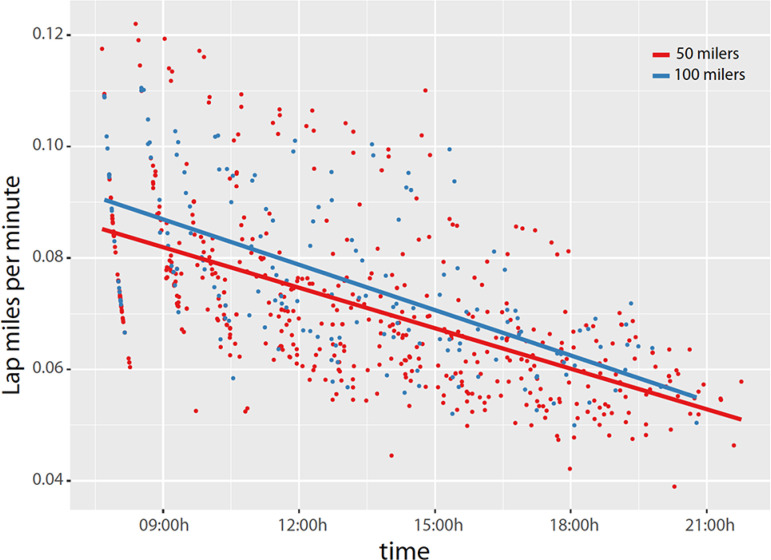
(A) Linear fit to account for the trending of a homeostatic influence: the slowing-down effect. All ultramarthoners slowed as time passed, and 100 milers (blue) were slightly faster compared to 50 milers (red).

**Figure 3:**
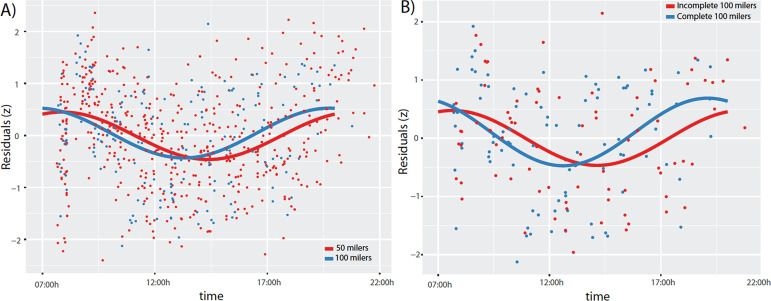
Cosinor fit to account for time-dependent differences in individual performance/speed for the first 50 miles of the race for (A) all 50- and 100-milers, and (B) only 100 miles. All ultramarathoners slowed in the early afternoon but sped up in the early evening. The acrophase of 100-mile runners (blue) was phase-advanced compared to 50-milers (red; A). The acrophase of runners who completed the 100 miles (blue, n=9) was phase-advanced compared to those who could not complete the 100 miles (red, n=8; B).

These data are represented in Figures 2 and 3 in order to determine if race pacing could predict future performance during a single-effort ultramarathon. Linear models were used to explain the linear trend and drop in speed for each individual for the first 50 miles. In the next step, the residuals of these models were then normalized and used for the cosinor model with period of 12 hours. Then we used the package “cosinor” in R program and used “*test_cosinor*” function with a period of 12h. Given a time and performance variables along with optional covariates, this function performs a Wald test to compare the groups having binomial values. In our analysis GROUP covariate consisted of 2-levels, 50 milers and 100 milers.

### Protocol approval and informed consent

Protocol approved by the Institutional Review Board of Indian River State College. A collaborative research agreement between Indian River State College and the Walter Reed Army Institute of Research permitted for analyses of de-identified data collected by approved research investigators and staff of Indian River State College per Department of Defense regulations. Informed consent was provided at host hotel of race site the night prior and prior to the race start time. Race data was not de-identified as it was publicly available.

## RESULTS

### Sleep, training and cardiovascular fitness/adaptation profiles of ultramarathoners (Fort Clinch and Florida Keys)

Data are summarized in Table 1. We broadly aimed to determine if runners completing 100 miles adopted different training and sleeping habits compared to 50-miler runners, or if sleep and training habits were generally similar for all ultramarathoners (50 or 100 miles). All runners (from the Florida spring race circuit) reported 6.8 + 0.2h of nighttime sleep during training (50- vs. 100-miles; *p*=0.56, Pearson Chi-Square; n=83, n=35 for 50-mile). On race day, nighttime sleep was self-reported at 6.1 + 0.2h. There were no differences in self-reported sleep quality between training days and rest days (*p*=0.54, two-way, two-tailed ANOVA). To broadly determine inter-individual variation in sleep need (for anabolic recovery) and daytime sleepiness, we asked runners if they napped. Although the rationale for napping was not known, we did observe differences between 100-miler and 50-miler runners. 38% of 100-miler runners routinely took naps, averaging 44.2 + 6.0 min (n=48). No 50-miler runner reported napping (n=35). To broadly determine differences in sleep efficiency with race duration, we found that self-reporting of sleep disturbances -- prolonged mid-night awakenings (> 20 min), restlessness, and night sweats -- were ~2-fold greater in 100-miler runners compared to 50-miler runners (93% vs. 50%; *p*=0.03, Pearson Chi-Square). 11.5% of runners used sleep aids (50- vs. 100-miles; *p*=0.70, Pearson Chi-Square) compared to 2.8% in the general population (Center for Disease Control, 2018).

We also aimed to identify if ultramarathons pre-select for early risers given time demands of training. Although pre-selection via chronotype cannot be inferred from our present assessment, these data did show likely influence of work-social constraints on pre-selection for early risers (for training). In our sample population, 46.2% of runners woke up before 07:00h. 19.2% of ultra-runners woke up after 09:00h. 42.3% trained in the early morning, starting before 07:00h. 26.9% trained in the evening after 17:00h. There was no difference between 50- and 100-milers for rise time or training preference (*p*=0.45 (rise time); *p*=0.15 (training), Pearson Chi-square). Lastly, we aimed to broadly characterized cardiovascular fitness/adaptation to > 24h exercise under sleep loss and thermal stress (85° F; 80% humidity, for both races) during the Florida spring race circuit. Given our criteria, only data for 100-miler finishers were included for analyses (n=127). We found a statistically significant but not clinically significant change (8.7% decrease in mmHg) in mean arterial pressure (MAP) five minutes before starting (91.3 + 1.0 mmHg, supine; 97.6 + 1.0 mmHg, standing; n=127) after finishing an ultramarathon (83.1 + 0.9 mmHg, supine; 83.3 + 1.0 mmHg, standing; n=127; *p*=0.0001 (supine); *p*=0.0001 (supine); paired t-tests).

### 24h human performance profiles (HPP) of ultramarathoners (Fort Clinch and Florida Keys)

Race pace and dropout rates were provided by race directors (n=257, in total) for general descriptive statistical analyses (mean *+* SE; SPSS ver. 21; *p*<0.05) of the Fort Clinch 50- and 100-miler Ultra (March 2016) and Florida Keys 50- and 100-miler Ultra (spring 2016). Race pace and dropout rates for the Fort Clinch 100-miler Ultra are summarized in Figure 1. The racecourse of the Fort Clinch Ultra (10-mile trail for 10 laps with 5-mile split times) allowed us to fully capture time-dependent differences in race pace defined here by the 24h HPPs. The Fort Clinch Ultra began at sunset (ZT 0) and sunrise was ~ 12h later at 1930 (ZT 12). The average finish time for 50 miles was 12.3 + 0.5h (n=50; 23, female): starting at sunrise and finishing near sunset. Average race pace was 12.4 + 0.3 min/mile for the first 10 miles (n=61; 28, female) and 15.8 + 0.5 min/mile for the last 10 miles (n=50; 23, female), slowing by ~ 1.5 min/mile across each consecutive 5-mile split of the 50 miles. The dropout rate was 18% or 5 females and 6 males. The average finish time for 100 miles was 25.2 + 3.8h (n=9 runners). Average race pace was 15.1 + 2.3 min/mile (Figure 1; n=21, 4 female). Prior to sunset (ZT 0 - ZT 12), average race pace was 13.7 + 0.6 min/mile. After sunset, runners slowed down by 35.9% (relative to ZT 0 - ZT 12). The slowest 5 miles were in the middle of the night (~ ZT 20; 20.8 + 3.2 min/mile; Figure 1). In laboratory studies, ZT 20 aligns with predicted circadian nadirs in human alertness and core body temperature^(17,18 )^but future research is required to better determine a circadian-dependent influence in field studies of human performance spanning > 24h such as reported here. At sunrise the next day (ZT 0 v.2; Figure 1), race pace recovered to the overall average of 15.3 + 1.0 min/mile. 25% of 100-milers dropped out 50 miles into the race. The dropout rate reached as high as 57% by 75 miles, occurring at ZT 20 when race pace was slowest. No runner dropped out past 75 miles (n=9, 2 females).

For the Florida Keys Ultra run along highway US-1, average finish time for 100 miles was 26.0 + 0.4h (n=122). Unfortunately, split times (per distance) were disproportionately tracked along the Florida Keys Ultra; race splits were logged every ~ 5 miles, ~ 10 miles, or ~14 miles, preventing us from constructing *clean* > 24h HPPs and performance predictions through cosinor fits (see below). However, we were still able to identify a meaningful relationship between race performance, distance, and time-of-day: the first 14-mile split (25 miles into 100 miles) was nearly an hour faster (3.6 + 0.1h) than the second 14-mile split (75 miles into 100 miles (4.5 + 0.3h; *p*=0.02; one-way ANOVA). Independent of race distance, the second 14-mile split coincided with ZT 20 (compared to ZT 6 of the first split).

### Performance prediction models of ultramarathoners (Fort Clinch)

In order to predict the ability to finish a 100-miler ultramarathon and to determine performance attributes that separate a 50-miler versus 100-miler ultramarathoner, we used race times from the Fort Clinch race since there was equidistant recording of race performance compared to the Florida Keys. We first modeled each runner’s performance with a linear fit to account for the trending of a homeostatic influence: the slowing-down effect. The linear model [“lm(lap mile per min~ time*GROUP, data=runners)”] indicated that all ultramarathoners slowed down as time passed (time, t=-10.157, *p*<0.01); an example of homeostatic load. In general, all 100 milers were slightly faster compared to 50 milers (t=-2.653, t=*p*<0.01; Figure 2). In order to detect temporal changes in performance/speed (miles per minutes), the normalized values of the individual residuals found after the initial linear model fit were then introduced in a cosinor-based model with a period of 12h. First, we found that all ultramarathoners slowed in the early afternoon, but sped up in the early evening. Second, we tested whether the acrophase or the amplitude of the cosinor fit for the 50-milers compared to 100-milers were statistically significant. The acrophase of 100-mile runners was phase-advanced compared to 50-milers (Wald=-2.36, *p*<0.05, 95% CIs [-0.85, -0.08]), meaning the 100-milers slowed earlier (late morning) and sped up earlier (mid-afternoon; Figure 3A). Amplitude did not differ (Wald=1.21, *p*=0.227). Most notably, we found that the runners who were able to complete the 100 miles slowed down earlier and sped up earlier (phase-advanced acrophase; n=9) compared to those who could not complete the 100 miles (n=8; (Wald=-2.13, *p*<0.05, 95% CIs [-1.24, -0.05]); Figure 3B). Amplitude did not differ (Wald=-1.09, *p*=0.277).

## DISCUSSION

At our research camps at single-effort ultramarathons in Florida (Fort Clinch Ultras and Florida Keys Ultras), we were able to broadly dissect time-dependent patterns of human endurance capabilities under high homeostatic load (total sleep deprivation/sleep loss). Our data are summarized in > 24h human performance profiles (HPP) that take race performance across time and distance into account. The strengths of our field study are: (a) we were able to create > 24h HPPs of endurance capabilities through minimal intervention of ultramarathon operations, ensuring that our data were ecologically valid; and (b) capitalized on the racing strategy of running through the night in order to create/control for total sleep deprivation/sleep loss. Some limitations of our study are that we did not use the full versions of self-reported sleep quality (Pittsburgh Sleep Quality Index) and chronotype questionnaires (Morning-Evening Questionnaire) for our assessment of self-reported sleep and training habits of ultramarathoners. Therefore, chronotype cannot be inferred in the present study. However, we were able to broadly capture adopted training times and general self-reported sleep behavior in ultramarathoners which is a novel concept. We also acknowledge this is one of few field studies of extreme human performance under total sleep deprivation. Our 24h HPPs of ultramarathoners allowed us to identify time-dependent predictions on overall performance under minimal sleep, warranting the ultra-marathon athlete as a unique demographic for future study of sleep and chronobiological relationships in the real world.

To begin, we macroscopically captured sleep and training habits and cardiovascular fitness/adaptation in > 80 ultramarathoners from two ultramarathons held during the late sub-tropical spring (85° F; 80% humidity, for both races). Our intent was several-fold. First, we aimed to determine if our assessments aligned with the recent sleep strategies of ultramarathoners described in Martin et al.^14^. Second, we wanted to examine the extent of anabolic recovery through sleep in response to extreme exercise. That is, could we determine greater sleep drive (i.e., hours) in 100-miler runners compared to 50-miler runners. Third, we broadly aimed to examine if ultramarathons pre-select for early risers (likely due to work-social constraints since pre-selection via chronotype cannot be inferred from our present assessment). Despite some limitations, our rationale was based on recent data showing that early risers (independent of athletic ability) have higher pain tolerances and better thermoregulatory control compared to late risers (independent of athletic ability)^10,17^.

In general, self-reported sleep in our study was < 1h under self-reported sleep of ultramarathoners in Martin et al.^14^. Unlike Martin et al.^14^, ultramarathoners in our study did not sleep extend during training or in preparation for a race; we found that self-reported sleep was similar for training days versus rest days. Also, self-reported sleep was 0.7h shorter the night before the race (rather than sleep extended) compared to training days. However, similar to Martin et al.^14^, we found that 38% of 100-miler runners napped (compared to ~ 50% in Martin et al.^14^). We also found differences in rates of self-reported napping (although rationale for napping is not known) based on distance run. 38% of 100-miler runners napped. 0% of 50-miler runners napped. These data lend credence to the idea that a higher physiological “set point” for sleep need (i.e., anabolic recovery) is required with increasing physical demands. Increased napping in 100-miler runners could also be due to 2-fold more sleep disturbances, including prolonged mid-night awakenings (> 20 min), restlessness, and night sweats, in 100-miler runners compared to 50-miler runners. Increased sleep disturbances with training distance (100 vs. 50 miles) can also be argued to be physiological evidence for overtraining.

We also found that intra-individual differences in preferred bedtime largely aligned with intra-individual differences in preferred training time. Nearly 50% of the ultramarathoners trained early in the morning likely due to work-social constraints but these data warrant future research as to whether ultramarathoners are indeed early morning chronotypes. Given the present data and previous reports of Martin et al.^14^, the next step will be to objectively determine if ultramarathoners are sleep-efficient or sleep-deficient, possibly through wrist-worn actigraphy and polysomnography, matched against functional outcomes: neurobehavioral performance. Essentially, it will be important to determine if: (a) ultra-runners have undergone biochemical re-wiring to adapt to less sleep-an example of adaptive homeostasis -- or ‘transient expansion (or contraction) of the homeostatic range in response to sub-toxic, non-damaging events’^6^*;* and (b) ultra-runners have genetic markers that confer resilience to total sleep deprivation.

Lastly, we observed that the human body quickly adapts to extreme exercise and total sleep deprivation (driven by the racing strategy of running through the night in order to finish an ultramarathon) through measurements of mean arterial pressure. In general, we found that mean arterial pressure (43.2 + 12.5 years) only varied by only 8% between the start and finish of a 100-mile race. Cardiovascular health is under circadian control^18^ and is impacted by both acute and chronic sleep loss in healthy individuals^2^. Given that our MAP assessment was likely more influenced by homeostatic load (> 24h exercise and wakefulness) compared to circadian load (MAP assessment pre- and post-race occurred at a similar time of day), we were able to provide evidence for quick adaptation to the demands of finishing an ultramarathon. Our results also aligned with a previous study of hemodynamic alteration after an ultramarathon^20^. In this study, blood pressure was not only lower post-race (relative to pre-race levels), but beyond our study, the researchers determined that diastolic blood pressure predicted the ability to finish.

Our main findings generated from the > 24h HPPs also lend credence to time-dependent regulation of human exercise capacities and outcomes^3,7,16^. There are several studies showing the time at which athletes compete and train can influence win-loss records^3^, injuries^3^, and individual performance^7,16^. In general, athletes perform better and are less likely to get injured in the late biological morning^3^ and late evening^3,7,16^, driven, in part, by rhythms of cortisol and norepinephrine tone^16^. Early morning performance can be raised to late morning or evening levels through stimulant supplementation^16^. Thus, these past studies may possibly be the basis for the performance “pushes” post-dusk and dawn in the present study but requires additional study of neuroendocrine factors. Second, although this is not the first field study in ultra-endurance athletes^1,9,11,14,19,20^ this is the first field study to map the 24h dynamics of human ultra-endurance capabilities. In general, we found time-dependent patterns of ultramarathon performance at the level of the individual (Figures 2 and 3) and group (Figure 1) level. Our cosinor analysis revealed a distinctive rhythm used to predict end point performance in that runners who slowed down earlier (late morning/noon) and sped up earlier (in the afternoon) were more likely to complete a 100-mile versus 50-mile race, and also predictive of the ability to finish a 100-mile race versus dropping out (Figures 2 and 3).

A secondary point to consider in generating our > 24h HPPs is the dropout rate. If an ultramarathoner chose to run past 50 miles, the dropout rate was highest in the middle of the night (ZT 20) when human alertness are predicted to be most vulnerable to time-driven physiology^5,15^. Dropouts were few past 75 miles, indicating some level of psychological motivation of “almost being done.” While we cannot exclude additional physiological and environmental influences on the ability to finish an ultramarathon, including nutritional strategies adopted across the race as reported previously^19^, this study is the first, to the best of our knowledge, to consider time-dependent predictors of the ability to finish a single-effort ultramarathon.

To conclude, our > 24h HPPs of ultramarathoners first reported here will enable us better understand the substrates and mechanisms of resiliency in extreme human conditions. These data are also critical for military operations. Ultramarathoners can provide biomarker discovery with the ability to inform how military personnel respond and adapt to insufficient sleep and environmental stress inherent of combat operations. Beyond combat operations, these data are valuable for high-performers in regards to determining when someone performs well or poorly with respect to time of day and/or require a countermeasure (e.g., caffeine) in order to return performance to optimal levels. These data also lend credence to re-defining fatigue and situational awareness in extreme environments, and offer a new perspective for tracking and managing physiological statuses under extreme stress as well as present the ultramarathoners as a unique demographic for future studies of sleep and chronobiological relationships in the real world.
